# Sleep spindle and K-complex detection using tunable Q-factor wavelet transform and morphological component analysis

**DOI:** 10.3389/fnhum.2015.00414

**Published:** 2015-07-28

**Authors:** Tarek Lajnef, Sahbi Chaibi, Jean-Baptiste Eichenlaub, Perrine M. Ruby, Pierre-Emmanuel Aguera, Mounir Samet, Abdennaceur Kachouri, Karim Jerbi

**Affiliations:** ^1^LETI Lab, Sfax National Engineering School, University of SfaxSfax, Tunisia; ^2^Department of Neurology, Massachusetts General Hospital, Harvard Medical SchoolBoston, MA, USA; ^3^DYCOG Lab, Lyon Neuroscience Research Center, INSERM U1028, UMR 5292, University Lyon ILyon, France; ^4^Electrical Engineering Department, Higher Institute of Industrial Systems of Gabes, University of GabesGabes, Tunisia; ^5^Psychology Department, University of MontrealMontreal, QC, Canada

**Keywords:** sleep, spindles, K-complex, automatic detection, electroencephalography (EEG), tunable Q-factor wavelet transform (TQWT), morphological component analysis (MCA), neural oscillations

## Abstract

A novel framework for joint detection of sleep spindles and K-complex events, two hallmarks of sleep stage S2, is proposed. Sleep electroencephalography (EEG) signals are split into oscillatory (spindles) and transient (K-complex) components. This decomposition is conveniently achieved by applying morphological component analysis (MCA) to a sparse representation of EEG segments obtained by the recently introduced discrete tunable Q-factor wavelet transform (TQWT). Tuning the Q-factor provides a convenient and elegant tool to naturally decompose the signal into an oscillatory and a transient component. The actual detection step relies on thresholding (i) the transient component to reveal K-complexes and (ii) the time-frequency representation of the oscillatory component to identify sleep spindles. Optimal thresholds are derived from ROC-like curves (sensitivity vs. FDR) on training sets and the performance of the method is assessed on test data sets. We assessed the performance of our method using full-night sleep EEG data we collected from 14 participants. In comparison to visual scoring (Expert 1), the proposed method detected spindles with a sensitivity of 83.18% and false discovery rate (FDR) of 39%, while K-complexes were detected with a sensitivity of 81.57% and an FDR of 29.54%. Similar performances were obtained when using a second expert as benchmark. In addition, when the TQWT and MCA steps were excluded from the pipeline the detection sensitivities dropped down to 70% for spindles and to 76.97% for K-complexes, while the FDR rose up to 43.62 and 49.09%, respectively. Finally, we also evaluated the performance of the proposed method on a set of publicly available sleep EEG recordings. Overall, the results we obtained suggest that the TQWT-MCA method may be a valuable alternative to existing spindle and K-complex detection methods. Paths for improvements and further validations with large-scale standard open-access benchmarking data sets are discussed.

## Introduction

We spend about one third of our lives sleeping. Luckily, and as might be expected of an efficient organism, the time we spend sleeping is not wasted idling. Sleep plays a functional role mediating a range of cognitive processes including learning and memory consolidation (Maquet, 2001; Walker and Stickgold, 2004; Diekelmann and Born, 2010; Fogel et al., 2012; Albouy et al., 2013; Rasch and Born, 2013; Stickgold and Walker, 2013; Alger et al., 2014; Vorster and Born, 2015), problem solving (Cai et al., 2009), sensory processing (Bastuji et al., 2002; Perrin et al., 2002; Ruby et al., 2013a; Kouider et al., 2014) and dreaming (Nielsen and Levin, 2007; Hobson, 2009; Nir and Tononi, 2010; Blagrove et al., 2013; Ruby et al., 2013b; Eichenlaub et al., 2014a,b). Sleep disorders, as well as the mere lack of sleep, can have serious effects on our health, both by deteriorating the proper function of sleep-related brain processes and indirectly by being a risk factor for conditions such as weight gain, hypertension and diabetes (Anderson, 2015). The utmost importance of a good night's sleep is therefore unquestionable. However, many questions related to the mechanisms and role of the numerous electrophysiological signatures of sleep are still outstanding. The standard approach to monitor sleep is the use of Polysomnography (PSG) which combines multiple physiological recordings including electroencephalogram (EEG), electromyogram (EMG), electrocardiogram (ECG), and electrooculogram (EOG). In addition to be being a central diagnosis tool for a range of sleep disorders (such as narcolepsy, idiopathic hypersomnia and sleep apnea), PSG is a valuable tool for sleep research performed in healthy individuals. In particular, the analysis of sleep EEG signals helps us understand its neurophysiological basis and functional role. Macro and micro-structures are present in sleep signals at various temporal scales. Macro structure analysis often refers to sleep staging, i.e., the segmentation of brain signals into 20 s or 30 s-long periods that represent different sleep stages, each with distinct cerebral signatures. On the other hand, micro structure analyses of brain signals during sleep consists of detecting short-lived microscopic events often considered to be hallmarks of specific sleep stages and of sleep-related cognitive processes, as well as potential signs of sleep anomalies. K-complexes and sleep spindles are among the most prominent micro-events studied in sleep studies, not only for their importance in sleep stage scoring (as they predominantly occur during S2 sleep stage), but also for their importance in the diagnosis of sleep disorders and the exploration of the functional role of sleep.

According to the American Academy of Sleep Medicine (AASM) (Iber et al., 2007), Sleep spindles are defined as: “A train of distinct waves having a frequency of 11–16 Hz with a duration ≥0.5 s, usually maximal in amplitude over central brain regions.” These waveforms, which are controlled by thalamo-cortical loops (e.g., Steriade, 2003, 2005; Barthó et al., 2014), are the subject of an active area of investigation that seeks to understand the mechanisms and functions of the sleeping brain. Numerous studies have shown that sleep spindles have an important role in memory consolidation during sleep (Schabus et al., 2004; Morin et al., 2008; Diekelmann et al., 2009; Diekelmann and Born, 2010; Barakat et al., 2011; Fogel et al., 2014; Lafortune et al., 2014). Moreover, sleep spindle characteristics undergo age-related changes (e.g., Seeck-Hirschner et al., 2012; Martin et al., 2013). Other studies suggest that sleep spindles are clinically important given that alterations in their density (number per minute) may be a symptom of neurological disorders such as dementia (e.g., Ktonas et al., 2009, 2014; Latreille et al., 2015), schizophrenia (e.g., Ferrarelli et al., 2010; Ferrarelli and Tononi, 2011), depression (Riemann et al., 2001), stroke recovery, mental retardation, and sleep disorders (De Gennaro and Ferrara, 2003).

K-complexes are defined by the AASM as “A well delineated negative sharp wave immediately followed by a positive component with a total duration ≥0.5 s, typically maximal at frontal electrodes” (Iber et al., 2007). The precise role of K-complexes in sleep is still a matter of debate. Some studies consider them as an arousal response, since they are often followed by micro-awakenings (Halász, 2005). Others give K-complexes a sleep “protection” function (Jahnke et al., 2012). Single-unit recordings during human sleep suggest that K-complexes may represent isolated down-states (Cash et al., 2009).

The ability to reliably detect the occurrence of sleep spindles and K-complexes in EEG recordings is therefore of major importance in a wide range of sleep investigations, ranging from basic research to clinical applications. Visual annotation of sleep spindles and K-complexes is tedious, time consuming, subjective and prone to human errors. The inter-agreement between multiple scorers (for spindles and K-complex visual marking) reported in the literature is relatively low (Zygierewicz et al., 1999; Devuyst et al., 2010; Warby et al., 2014). Therefore, as in sleep staging (e.g., O'Reilly et al., 2014; Lajnef et al., 2015), automatic or semi-automatic identification procedures are of great utility for the detection of sleep spindles and K-complexes. Approaches based on band-pass filtering and thresholding have been proposed for both spindles and K-complex detection (e.g., Huupponen et al., 2000; Devuyst et al., 2010). Template-based filtering using matching pursuit methods has also been used proposed (e.g., Schönwald et al., 2006). Other filtering approaches based on continuous wavelet transforms (CWTs) have also been explored (Erdamar et al., 2012). Moreover, signal classification methods have been used to detect K-complexes or spindles, for instance, using artificial neural networks (ANN) (e.g., Günes et al., 2011), Support Vector Machines (SVMs) (e.g., Acir and Güzelis, 2004) and decision-trees (Duman et al., 2009). Interestingly, only a handful of studies have investigated the detection of K-complex and spindles simultaneously using a common methodological framework (Jobert et al., 1992; Koley and Dey, 2012; Jaleel et al., 2013; Camilleri et al., 2014; Parekh et al., 2015).

In this study we propose a framework for joint spindle and K-complex detection. The proposed method combines a recently introduced discrete wavelet transform (DWT) known as the Tunable Q-factor Wavelet Transform (TQWT) (Selesnick, 2011a) with Morphological Component Analysis (MCA). This combination provides a natural and efficient way to decompose the EEG signal into transient (K-complex) and oscillatory (spindle) components. The results we obtain with full-night sleep EEG recordings from 14 participants demonstrate the utility and added-valued of the proposed method. Our method also performed well when compared with a standard spindle detection method and when applied to a publicly available spindle and K-complex data set.

## Materials and methods

### K-complex and sleep spindle detection method overview

**Figure 1:**
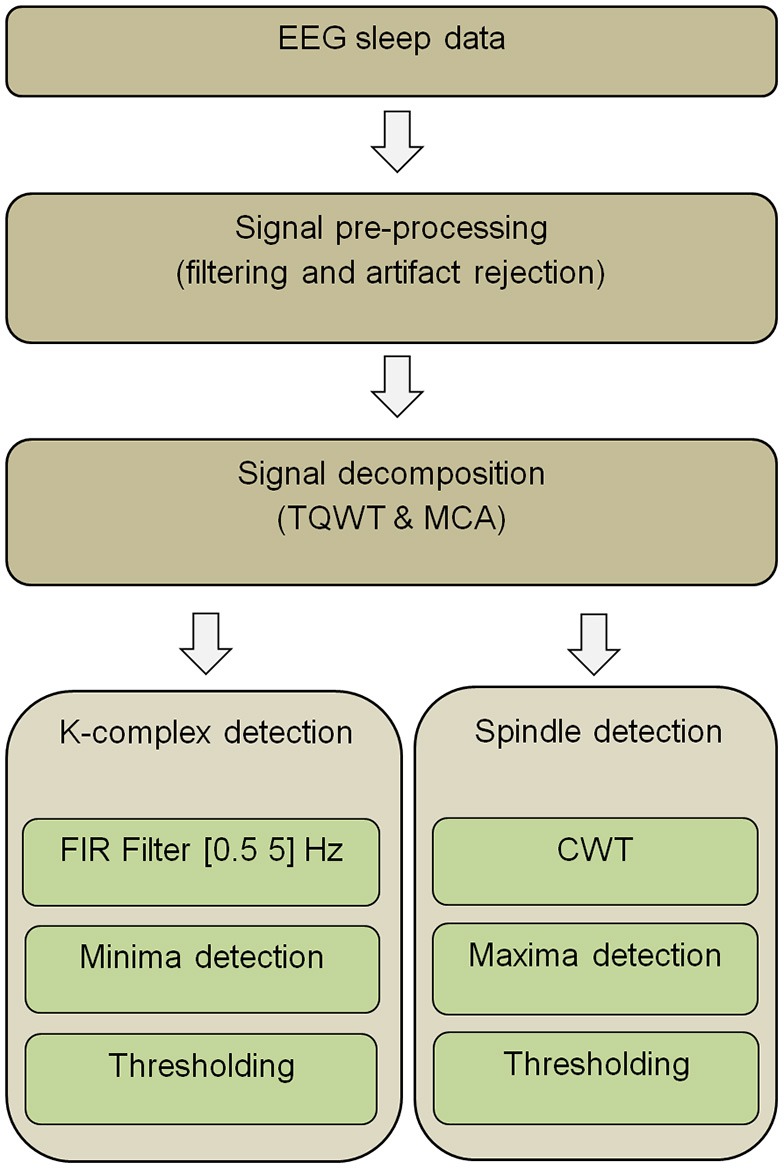
**Overview of the proposed EEG data analysis pipeline for K-complex and sleep spindle detection (Abbreviations: TQWT, Tunable Q-Factor Wavelet Transform; MCA, Morphological component analysis; CWT, Continuous Wavelet Transform; FIR, Finite Impulse Response)**.

The main steps of the K-complex and spindle detection pipeline are presented in Figure 1. First, EEG segments are filtered so as to reduce the effect of potential artifacts. The filtered signals are then decomposed into oscillatory and transient components by combining a TQWT with MCA. Next, applying FIR filtering to the transient component unveils K-complex events, while applying a CWT to the oscillatory component unravels spindle events. The appropriate detection thresholds that need to be used in the final step are determined by plotting sensitivity against false discovery rate (FDR) for a range of potential thresholds [an approach akin to Receiver Operating Characteristic (ROC) curves] calculated from a subset of the data (training set). The ROC-like curves are obtained by repeatedly measuring sensitivity and FDR while varying the threshold parameters and using expert visual marking of K-complexes and spindles as ground truth. The steps that make up the proposed pipeline (Figure 1) are described in detail the next sections.

### EEG sleep recordings

#### Data acquisition

The EEG data used in this study was collected from 14 healthy subjects aged 29.2 ± 8 years, all recorded at the DyCog Lab of the Lyon Neuroscience Research Center (CRNL, Lyon, France) with a sampling frequency of 1000 Hz. The data acquisition was part of a research program exploring cognition during sleep (Eichenlaub et al., 2012, 2014b; Ruby et al., 2013a,b). The EEG component of the polysomnography recordings across the 14 subjects were visually scored by an expert in successive windows of 30-s using the R and K guidelines (Rechtschaffen and Kales, 1968). The sleep staging step here gave us the possibility to choose to run our detection pipeline (a) exclusively on S2 sleep segments, or (b) on all sleep stages (as would be the case in the absence of sleep scoring). In other words, sleep staging is not a required pre-processing step for the detection method proposed here. Unless otherwise stated, all the analyses described were based on the standard EEG C3 channel.

#### Splitting the data into training and test sets

To evaluate the performance of the detection procedure, we divided the data base into a training set (used to derive optimal thresholds via ROC-like curves) and a test set used to compute the performance of the method. Thirty S2 segments and 15 non-S2 segments were randomly selected from the data of each of the 14 individuals (i.e., 630 sleep EEG data segments in total: 420 S2 segments and 210 non-S2 segments). This ensured a balanced representation of data from across all subjects. Note, that our emphasis on S2 stems from the fact that it is the sleep stage of primary interest for the detection of spindles and K-complexes. As a general rule, we used equally sized training and test sets (210 segments for testing and 210 segments for training). The training and associated test sets consisted either of S2 segments only (scenario 1), or of a mixture of S2 and non-S2 segments (scenario 2). Note that in this second case, the test and training sets contained 105 S2 and 105 non-S2 segments. As we spend approximately half our sleep in stage 2 (Carskadon and Dement, 2011), this proportion was representative of using random sampling of sleep segments. In addition, for practical purposes, we also explored the effect of reducing the size of the training set to evaluate minimal training requirements (scenario 3).

#### Signal preprocessing and visual annotation of microstructures

The presence of various artifacts in sleep EEG adversely affects both visual and automatic detection of spindles and K-complexes. The EEG signals were therefore band-pass filtered with low and high cutoff frequencies at 0.2 and at 40 Hz, respectively. This was followed by visual inspection in search of potential remaining artifacts. In addition, visual annotation of K-complex and spindles on the EEG traces was independently performed by two experts and used as two alternative benchmarks. To facilitate this procedure, we designed a graphical user interface (GUI), which was used by our experts to visually explore the EEG data segments and identify K-complex and spindles events. The results of the visual detection were saved to two separate text files containing segment number, start and end times/sample for each event. For example, the visual annotation by Expert 1 of the 420 segments of S2 sleep across all subjects led to the identification of 437 Spindles and 293 K-complexes (see details in Table 1).

**Table 1:** **Example of visual annotation results by Expert 1 based on 420 S2 segments**.

	**Spindles**	**K-complexes**
Number of segments with	244	199
Number of segments without	176	221
Number of detected events	437	293

### EEG signal decomposition using TQWT and MCA

K-complexes and spindles are microstructures that are morphologically different. One major difference is that K-complexes are transient while spindles are oscillatory. To exploit this distinction, we set out to combine the recently introduced TQWT discrete wavelet with MCA in order to conveniently decompose any given EEG segment into two signals; a K-complex channel and a spindle channel. The decomposition via TQWT and MCA is described below.

#### Tunable Q-factor wavelet transform (TQWT)

TQWT is a flexible fully DWT that was recently introduced by Selesnick (2011a,c), for which the Q-factor of the wavelet is easily tuned and adapted to the signal being investigated. In principle, a high Q-factor transform is suitable for oscillatory signals, whereas transient signals are modeled using low Q-factor wavelets. Like the dyadic DWT, TQWT consists of iteratively applying two-channel filter bank, where the low-pass output of each filter bank is the input to the next filter bank. A sub-band is then defined as the output signal of each high pass filter. Considering J the number of filter banks, there will be J + 1 sub-bands, i.e., J sub-bands coming from the high-pass filter output signal of each filter bank and the low-pass filter output signal of the final filter bank. At each level, the generation of low-pass sub-band *C*^*j*^[*n*] uses a low-pass filter $H_{0}^{j}\left(w\right)$ followed by low-pass (LP) scaling α, and similarly the generation of high-pass sub-band d_*j*_[n] uses a high-pass $H_{1}^{j}\left(w\right)$ and high-pass (HP) scaling β. $H_{0}^{j}\left(w\right)$ and $H_{1}^{j}\left(w\right)$ are defined as follows (Selesnick, 2011a):

(1) $$H_{0}^{\left(j\right)}\left(w\right)=\{\begin{array}{c} \prod_{m\;=\;0}^{j\;-\;1}H_{0}\left(\frac{w}{\alpha^{m}}\right),\;\left|w\right|\leq \alpha^{j}\pi \\ 0,\;\alpha^{j}\pi <\left|w\right|<\pi \end{array}$$

and

(2) $$H_{1}^{\left(j\right)}\left(w\right)=\left\{ \begin{array}{l} H_{1}\left(\frac{w}{\alpha^{j\;-\;1}}\right)\prod_{m\;=\;0}^{j\;-\;2}H_{0}\left(\frac{w}{\alpha^{m}}\right),\\ \;\left(1-\beta \right)\alpha^{j\;-\;1}\leq \left|w\right|\leq \alpha^{j\;-\;1}\pi \\ 0,\;for\;others\;w\epsilon [-\pi ,\pi ]. \end{array}\right.$$

All main parameters were computed as described in the original study by Selesnick (2011a) and the user-manual of the TQWT toolbox (Selesnick, 2011b). Three key parameters that need to be set are the following:

- **Q-factor:** In the context of the present TQWT implementation, the Q-factor is theoretically defined as Q =(2−β)∕β. As it reflects the oscillatory behavior of the wavelet, the Q-factor can be set to fit the nature of the signal to which it is applied. In other words the parameter Q can be used to tune the wavelet function to the signal it seeks to model. The signals of interest here are sleep spindles and K-complex events. So to tune the TQWT wavelet toward spindles, we selected a *Q*-value that corresponds to the minimum number of cycles in a spindle burst. As the latter occur predominantly within 11–16 Hz frequency range with a minimum duration of 0.5 s, we chose *Q* = 5.5. In contrast, to tune the wavelet toward the K-complex component (one cycle) we chose *Q* = 1, as this provided a wavelet that closely models the shape of a transient wave.
- **Maximum number of levels (J**_*max*_**):** The selection of J_max_ depends on the length of the input signal (N) and the chosen filter scaling parameters (α and β) and is defined by the following equation: J_max_ = log(βN∕8) ∕log(1∕α) (Selesnick, 2011a).
- **Redundancy parameter (r):** The redundancy parameter r controls the excessive ringing in order to localize the wavelet in time without affecting its shape. Here, it's defined as: r = β∕(1−α). The specific value *r* = 3 has been previously recommended when processing biomedical signals (Selesnick, 2011a,b).

#### Morphological component analyze (MCA)

The goal of the MCA is to decompose a given signal x into two or more components on the basis of their sparse representation. In our case, MCA is used to decompose a given EEG signal x into an oscillatory component *x*_1_, and a transient signal *x*_2_, such that:$x=x_{1}+x_{2},\text{ where }x,\;x_{1},\;x_{2}\;\in R^{N}$. Most importantly, this decomposition is carried out using the TQWT transform (described above) as the sparse representation of x (Selesnick, 2011c). According to the MCA implementation using basis pursuit de-noising with dual Q-factors described in Selesnick (2011b), the sparse wavelets coefficients *w*_1_ and *w*_2_ associated respectively with x_1_ and x_2_ can be estimated via the minimization of the following function:

(3) $$\begin{array}{l} argmin_{w_{1},w_{2}}{\left\|x-\Phi_{1}^{*}\left(w_{1}\right)-\Phi_{2}^{*}\left(w_{2}\right)\right\|}_{2}^{2}+\sum_{j\;=\;1}^{J_{1}\text{+ 1}}\lambda_{1,\;j}||w_{1,\;j}|{|}_{1}\\ \;+\sum_{j\;=\;1}^{J2\text{+ 1}}\lambda_{2,\;j}||w_{2,\;j}|{|}_{1} \end{array}$$

**Figure 2:**
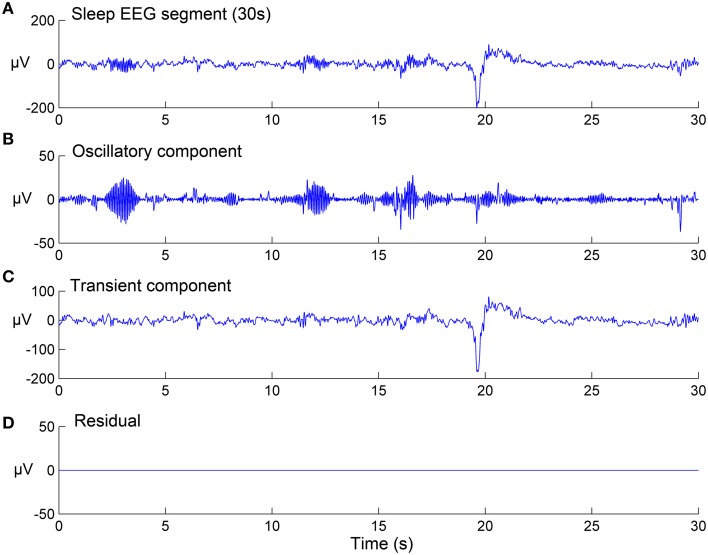
**Signal decomposition of a 30-s sleep EEG segment (A) into an oscillatory component (B) and a transient component (C) using TQWT-MCA method, with no residuals (D)**. See Section EEG Signal Decomposition using TQWT and MCA for method details.

Where Φ_1_ and Φ_2_ are two matrices of TQWT parameters: (Q_1_, r_1_, J_1_) and (Q_2_, r_2_, J_2_) respectively, *w*_1_ and *w*_2_ are vectors which contain the concatenation of the wavelet transform sub-bands, and λ_1, *j*_ and λ_2, *j*_ are the regularization parameters associated respectively with the two types of wavelets (They are two vectors of lengths J_1_ + 1 and J_2_ + 1, respectively). The sparse set of wavelet coefficients *w*_1_ and *w*_2_ are hence obtained, via the convergence of the objective function given by Equation (3). In the current study, the sparsity (few non-zero coefficients in *w*_1_ and *w*_2_ vectors) was achieved by setting the number of iterations for the convergence to 500. Next, the components x_1_ and x_2_ are estimated by: x_1_ = $\Phi_{1}^{*}w_{1}$ and x_2_ = $\Phi_{2}^{*}w_{2}$ (where $\Phi_{1}^{*}$ and $\Phi_{2}^{*}$ are the inverse TQWT matrices). Note that all parameters and variables described here were computed strictly as described in the original study by Selesnick (2011a) and user-manual of the TQWT toolbox (Selesnick, 2011b). Figure 2 shows the results of the TQWT-MCA decomposition applied to an illustrative 30-s EEG segment that contains three spindles and one K-complex. Panels B and C show the decomposition into selected oscillatory and transient components. The next step is to apply a detection procedure to identify the individual spindles and K-complex events from both components. The detection step for each is described in the next sections.

### Spindle detection

The oscillatory component obtained from the EEG decomposition procedure described above is used to detect the occurrence of sleep spindles. Applying a simple threshold directly to this signal would not be appropriate since spindles do not have an established range of amplitudes. Instead, we decided to detect the spindles by filtering the oscillatory component using a CWT.

#### Continuous wavelet transform (CWT)

**Figure 3:**
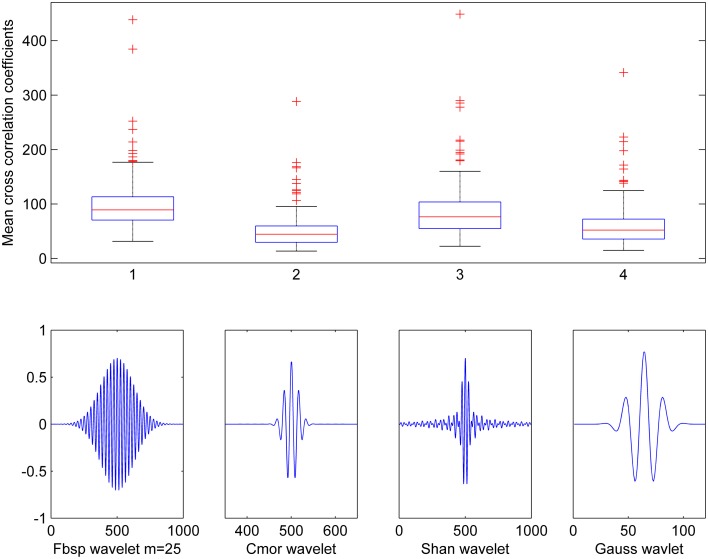
**Cross-correlation between various wavelet functions and spindles waveforms**. Results show mean cross-correlation between 210 spindle waveforms and five distinct wavelet functions: Frequency B-spline (Fbsp with *m* = 25), Complex Morlet wavelet (Cmor), Shannon wavelet (Shan), and Gaussian wavelet (Gauss).

To optimize the selection of the wavelet function to use in the CWT analysis, we computed the cross-correlation between several wavelet functions (Teolis, 1996) and the spindle waveforms present in the training data set. Based on visual inspection of similarity with the spindle waveform, we chose to test the following wavelet functions: complex frequency B-spline wavelets (Fbsp), complex Morlet wavelets (Cmor), complex Shannon wavelets (Shan), and Gaussian wavelets (Gauss). Figure 3 shows these individual wavelet functions as well as boxplots for the cross-correlation mean values obtained when using each one of them. Although the results were very close, Fbsp showed the highest maximal value (upper line of each box) and the highest median (red line) cross-correlation with the spindle waveforms. Therefore, we chose to use complex frequency B-spline wavelets which are defined as $bsp\left(t\right)=\sqrt{fb}\left[{sinc}^{m}\left(t.\frac{fb}{m}\right).e^{j2\pi f_{c}t}\right]$, where m is an integer parameter (m ≥ 1) that can be selected so as to ensure the best time-frequency resolution, fb is the bandwidth parameter and f_c_ is the wavelet center frequency. The CWT-based time-frequency maps computed throughout this study are based on this Fbsp wavelet function in the pre-defined frequency band of sleep spindles (i.e., 11–16 Hz).

#### Detection of local maxima and thresholding

To detect the occurrence of sleep spindles from the time-frequency (T-F) map of the oscillatory component, we first search for all local maxima by identifying T-F values that exceed those of all eight surrounding neighbors of any given value in the 2D time-frequency space (using a sliding window across the T-F two-dimensional space). Next, we apply a detection threshold to the obtained maxima in the T-F map. Selecting an optimal threshold is a critical step. We chose a procedure that determines the best threshold as the one that maximizes the difference between sensitivity (Sen) and FDR of spindle detection (Note that other options are of course possible and can easily be included in our framework). A practical way to achieve this goal is by using an ROC-like approach on a training data set. The concept is straight-forward: we compute the values of sensitivity and FDR of the detection method repeatedly as we gradually increase the threshold used in the last step. This procedure yields a curve that depicts how sensitivity and FDR co-vary as the detection threshold is changed. The optimal threshold is the one that maximizes the difference between sensitivity (ideally as high as possible) and FDR (ideally as low as possible). Note that the computation of FDR and sensitivity (see Section Performances Metrics) requires the use of some form of ground truth. Here we used expert visual marking as the benchmark. As our data was visually annotated (for K-complexes and spindles) by two experts, unless otherwise stated, we report all our results using, as ground truth, the annotation of each separately.

In summary, the optimal threshold derived from the “sensitivity vs. FDR” analysis is used when running the detection pipeline on the test data set. In order to evaluate the performance of the method, we compute once again sensitivity and FDR, but now only on the results obtained with the test set. The interested reader can find more details on such training procedures for instance in the appendix of Chander (2007).

### K-complex detection

Unlike sleep spindles, K-complex waveform is distinguishable from EEG background activity by “*a well delineated negative sharp wave*.” Therefore, our rationale was that applying a negative amplitude threshold on the transient components (derived from the TQWT and MCA procedure) could be a promising way to detect such events. However, in order to reduce the effect of some high frequency waveforms which generate local minima with amplitudes close to those of the K-complex (Devuyst et al., 2010), we first apply a band-pass FIR filter [0.5–5 Hz] to the transient component produced by TQWT and MCA step. Next, K-complexes are detected from the list of all local minima in each segment using an optimal threshold value. Note that we constrained the interval between two successive detected minima to be at least 2 s long to reduce risks of false detections. An EEG structure composed of multiple successive local amplitude peaks (such as delta waves) could in theory lead to the detection of a succession of transients and thus lead to the identification of successive K-complexes. This is only acceptable if the successive events are separated by at least 2 s, as that is approximately the minimal interval expected between two real K-complexes. The method used to derive the best threshold value to use here for K-complex identification is identical to the method described for threshold selection in the case of spindle detection: We use an ROC-like training procedure just as described in Section Detection of Local Maxima and Thresholding.

### Performances metrics

To compute the ROC-like curves used to derive detection thresholds (from the training set), and to evaluate the performance of our method (on the test set) we compute two basic metrics: the sensitivity (*Sen*) and *FDR* defined by Equations (4) and (5) respectively:

(4) $$Sen=\frac{TP}{TP+FN}$$

(5) $$FDR=\frac{FP}{FP+TP}$$

Where TP (true positive detections) are the events marked by the expert and correctly detected by our method, FN (false negative detections) are the events marked by the expert but not detected by the method and FP (false positive detections) represents the number of events detected by the method but which were not marked by the expert. Note that in detection contexts with strongly unbalanced occurrences of positive and negative cases, the ROC curve can provide an inadequate representation of the performance of a classifier (O'Reilly and Nielsen, 2013). This is the case here for the sleep EEG events we set out to detect because the continuous EEG segments consist predominantly of true negatives. This is why, instead of using standard ROC analysis, i.e., plotting sensitivity vs. false positive rate (or 1-specificity), we chose to depict sensitivity vs. FDR.

### Expert identification and inter-annotator agreement metrics

Two annotators visually identified all K-complexes and spindle events in our database. Unless otherwise stated all automatic detection results are evaluated against the annotation of Expert 1 and 2, independently. When evaluating the minimal number of training segments needed for our method (Section Impact of the Amount of Available Training Data on the Performance) and when exploring the results on a subject by subject basis (Section Performance of the Method in Individual Subjects) we restricted the analysis to the segments where Expert 1 and Expert 2 fully agreed (consensus). Inter-annotator agreement was assessed using two metrics: (i) percent agreement (portion of events on which raters compared to total number of events) and (ii) Cohen's kappa coefficient κ, a statistical measure of inter-annotator agreement that takes into account the agreement occurring by chance (Cohen, 1960).

## Results

The results of the proposed methodology are presented in the next sections as follows: First, we provide the results of the training step (ROC-based identification of optimal thresholds), followed by the performance of the method on test sleep data (S2 and non-S2). Next, we report also on the improvements achieved by using the optional adjustment step where the expert reviews (accepts/rejects) the false positive outputs of the method. We then explore the practical utility of the method by monitoring its performance as a function of training set size. Unless otherwise stated, we report all our results using, as ground truth, the annotation of each one of the two experts separately. This provides further insights into the robustness of the method.

### Detection of optimal threshold values (training phase)

**Figure 4:**
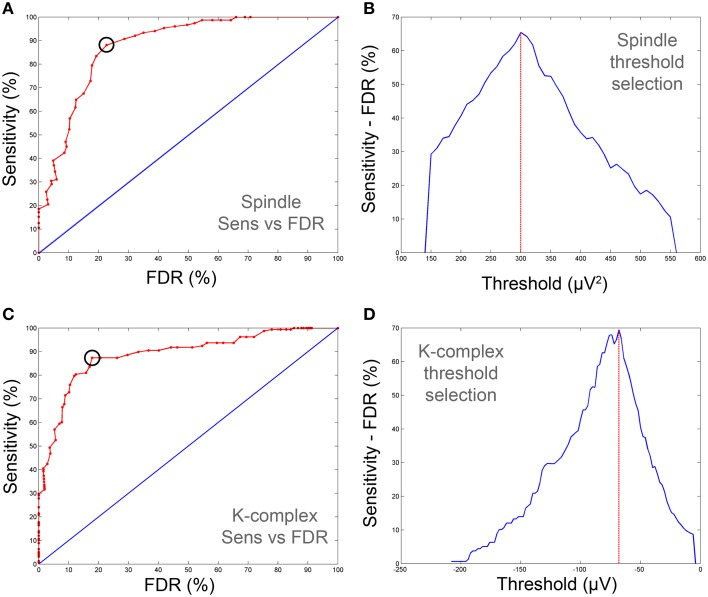
**Sensitivity-FDR plots used for the determination of optimal spindle and K-complex detection thresholds (on training data)**. **(A,C)** show ROC-like-curves of sensitivity vs. FDR for spindles and K-complex respectively. **(B,D)** depict the difference between sensitivity and FDR as the threshold is varied. The optimal thresholds were defined as those corresponding to maximum difference (vertical red line). The corresponding cut-off point is also depicted on **(A,C)** with black circles. Note that the sensitivity and FDR in the illustrative examples presented here are computed against consensus scoring (i.e., agreement between both scorers).

In the training phase, we used a subset of the data (training set) to derive “sensitivity vs. FDR” curves by evaluating sensitivity and false detection rates as we vary the detection threshold. Sensitivity and FDR were computed using 210 30-s EEG S2 data segments for threshold values that varied in steps of 10 μV^2^ for spindles and 2 μV for K-complexes (the unit reflects the fact that the thresholds are applied to time-frequency maps and voltages, respectively). The optimal threshold value, defined as the one that maximizes the difference between sensitivity and FDR, was determined from these curves and then used subsequently in the validation phase (i.e., using the test set). For spindle detection, this compromise in the training data was achieved by a threshold set to 290 μV^2^, yielding a sensitivity of 87.09% and an FDR of 45.68%. In the case of K-complex detection, a threshold value of −70 μV provided the best compromise, with a sensitivity of 78.72% and an FDR of 23.44%. The above results were obtained when using Expert 1 as benchmark. The results were very similar when relying on the annotation by Expert 2 as benchmark: For spindle detection, this compromise in the training data was achieved by a threshold set to 300 μV^2^, yielding a sensitivity of 83.45% and an FDR of 27.68%. In the case of K-complex detection, a threshold value of −70 μV provided the best compromise, with a sensitivity of 85.76% and an FDR of 32.22%. Figure 4 shows an example that illustrates the results of the training step and how the optimal threshold levels are determined. The identified thresholds are then used when applying the detection pipeline to the test segments (see next section). Throughout the paper, the training strategy was applied using visual scoring either by Expert 1, Expert 2 or by only using the data segments for which both experts fully agreed (consensus). Unless otherwise stated, we report the results of each analysis by providing the results obtained against Expert 1 and Expert 2 independently.

### Spindle and K-complex detection performance (test set)

**Figure 5:**
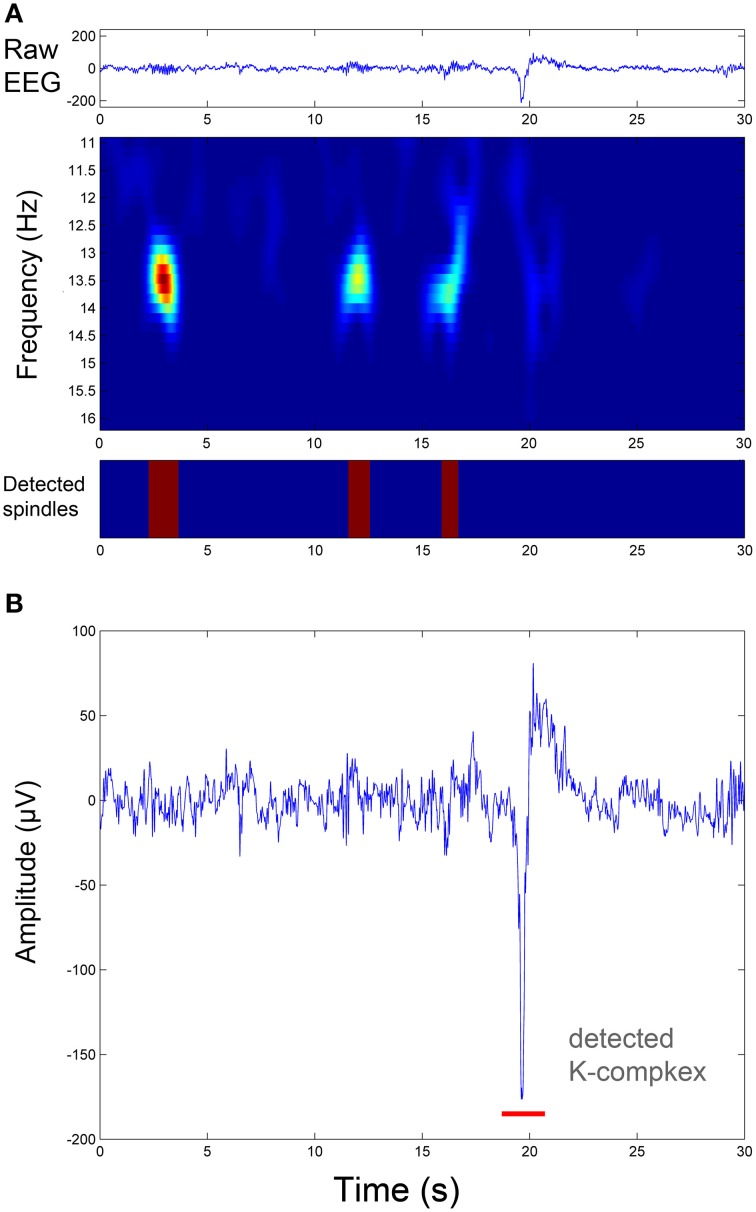
**Illustration of sleep spindle and K-complex detection in the same EEG segment shown in Figure 2**. **(A)** Spindle detection procedure: from the raw EEG signal (upper panel) to the time-frequency representation of the oscillatory component resulting from the TQWT-MCA decomposition (middle panel), to spindle identification (lower panel). **(B)** The associated K-complex detection in the same segment by thresholding the transient component resulting from the TQWT-MCA decomposition (see Figure 2).

To evaluate the performance of the pipeline and, in particular, assess the success of the threshold identification procedure, the spindle and K-complex specific thresholds identified in the training phase were then used to run the detection algorithm on previously unseen test segments. Figure 5 illustrates the detection procedure on the same sample sleep segment shown presented in Figure 2. The global results obtained for all 210 test EEG S2-sleep segments are shown in Table 2. The full analysis (training and testing) was repeated twice, each time using a different scorer as ground truth to explore the robustness of the procedure. The results indicate that the method proposed here yields a reasonably high sensitivity both for spindles (scorer 1: 83.18%, scorer 2: 81.57%) and K-complex (scorer 1: 81.57%, scorer 2: 85.25%). The FDR values for spindles reached 39% (scorer 1) and 19.66% (scorer 2), while the FDR for K-complex detection was 29.54% and 32.82% for scorers 1 and 2, respectively. Note that the inter-rater overall agreement was 77.85% (Cohen's kappa 0.64) and 63.33% (Cohen's kappa 0.51) for spindle and K-complex identification respectively. Table 2 also shows the method performance when applied exclusively to data segments for which both scorers agreed (100% inter-rater agreement, i.e., consensus scoring). In the case of spindle identification, this led to a sensitivity of 86.40% and an FDR of 29.22%.

**Table 2:** **Method performance (Sensitivity and FDR) obtained by applying the pipeline to the validation data set (test segments) for spindles and K-complexes detection**.

	**Sensitivity (%)**	**FDR (%)**
**SCORER 1**		
Spindle	83.18	39.00
K-complex	81.57	29.54
**SCORER 2**		
Spindle	83.10	19.66
K-complex	85.25	32.82
**SCORER 1 and 2 (AGREEMENT)**		
Spindle	86.40	29.22
K-complex	80.86	21.39

*Results are shown for scorer 1, scorer 2 and also for the case where only data with full agreement between the two scorers were used*.

### Performance comparison with and without TQWT and MCA

**Figure 6:**
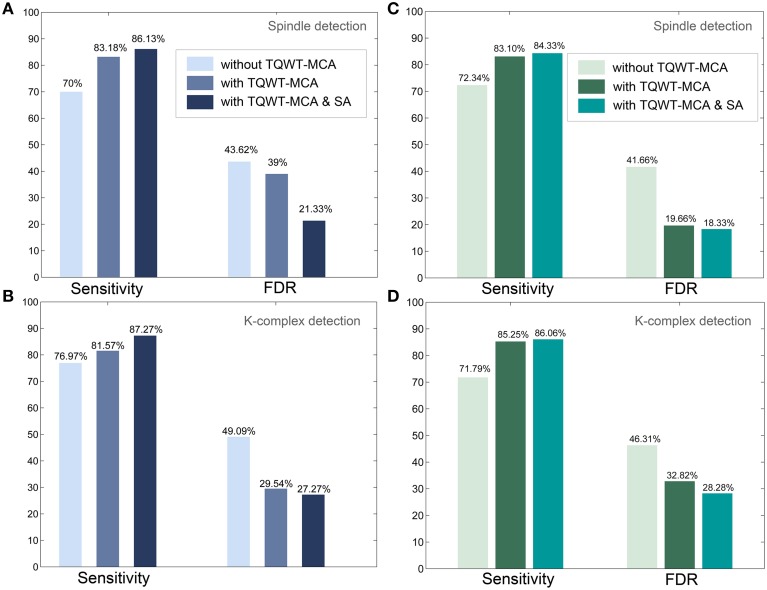
**Detection performances with and without the TQWT-MCA decomposition step and additional performance enhancement via scorer adjustment (SA). (A)** Sensitivity and FDR metrics for spindle detection (with scoring by Expert 1 used as ground truth), **(B)** Sensitivity and FDR metrics for K-complex detection (with scoring by Expert 1 used as ground truth). **(C,D)** Same as **(A,B)** respectively, but now using the scoring by Expert 2 as ground truth. The detection of spindles and K-complexes is enhanced by the use of TQWT-MCA and further improvements are obtained using the scorer adjustment approach.

How critical is the inclusion of the TQWT-MCA decomposition framework proposed here for the performance of the detection? To address this question we set out to evaluate the added-value of TQWT and MCA decomposition in the detection process. To this end, the entire pipeline was performed again on the same data set as above but this time with one notable difference: the TQWT and MCA steps were excluded from the method. In other words, instead of using oscillatory and transient components (i.e., the output of TQWT-MCA), the detection process started directly from raw EEG signals for K-complex identification, and directly from its CWT transform for spindle detection. Figure 6 compares the results obtained with and without the TQWT-MCA step. When using Expert 1 as ground truth, excluding the proposed decomposition led to a drop in sensitivity for spindle detection (from 83.18 down to 70%) and for K-complex detection (from 81.57 down to 76.97%). Deterioration was also observed in terms of increased false detections. The FDR values increased from 39 to 43.62% in spindles detection and rose from 29.54 to 49.09% for K-complex detection. The corresponding results obtained with Expert 2 as ground truth are comparable and are given in panels C and D of Figure 6. These findings quantify the specific added-value of the TQWT-MCA decomposition as a pre-processing step, as compared to direct detection on the raw EEG signal. In the discussion section, we further confirm these observations by comparing our method to another peak detection method previously published in the literature.

### Scoring adjustment based on expert review of false positives

We evaluated the potential performance enhancement that would be achieved by an additional (optional) step in which the false positive detections of our algorithm were presented to the expert scorer for review. This allowed the scorer to decide to accept or reject events detected by the algorithm but that he had initially not marked. A dedicated GUI was developed for this score adjustment (SA) procedure. After this process was carried out a new file with the adjusted score was created and the whole detection pipeline was repeated (i.e., including the training and validation processes). The performance enhancement obtained with the SA procedure is shown in Figure 6. As expected, sensitivity increased and FDR decreased, for K-complexes and spindles. The most prominent improvements were a drop in spindle FDR from 39 to 21.33% and an increase in K-complex sensitivity from 81.57 to 87.27%, when using the annotations of Expert 1 (Figures 6A,B). Similar results were obtained when comparing against annotations by Expert 2 (Figures 6C,D). Note that this semi-automatic step is not considered part of the proposed methodology, as it requires visual marking of the whole data set. Nevertheless, this analysis quantifies the impact of the subjective scoring, and provides an estimate of the performance that the method could provide if the scorer provides a more consistent visual marking.

### Stability of the proposed method with regards to sleep stages

The results presented above were obtained with EEG segments that were recorded during S2, the sleep stage where K-complex and spindles are most frequent. However, as indicated above, our method does not require sleep staging as a preliminary pre-processing step. The method is in theory equally valid for EEG segments from all sleep stages. We therefore also examined the performance of the detection algorithm by using 420 EEG segments including data from all sleep stages. Half of the segments were S2 (i.e., 210 segments) and the other half were non-S2 sleep (i.e., 210 segments). The 210 non-S2 segments were composed of: 126 REM segments, 42 SWS segments and 42 S1 segments. Note that these proportions were chosen to be close to the natural distribution (frequency of occurrence) of the various sleep stages across a typical night's sleep (Carskadon and Dement, 2011). The motivation behind this selection was to create training and test sets with compositions as close as possible to what one would get from a random sampling of sleep EEG epochs, i.e., without access to sleep stage information. Using equal number of events across sleep stages (or running our analysis separately for each sleep stage) was not feasible with the data at hand given that some of the sleep stages, in particular S1 and REM, contain a very low number of spindles and K-complexes.

Globally speaking, the results of this analysis (see Table 3) show a slight increase in sensitivity but comes at the expense of an increase in FDR. This is most likely due to the fact that the thresholds are better tuned to the more numerous S2 events. Note that also in this analysis we see a reasonable agreement between the results obtained when using each of the two scorers as ground-truth.

**Table 3:** **Method performance (Sensitivity and FDR) obtained by applying the pipeline to a validation data set (test segments) for spindle and K-complex detection that includes data from all sleep stages (S2 and non-S2 segments)**.

	**Sensitivity (%)**	**FDR (%)**
**EXPERT 1**		
Spindle	86.82	45.36
K-complex	80.23	37.27
**EXPERT 2**		
Spindle	85.05	32.19
K-complex	82.5	38.00

*Results are shown for both scorers. The performances obtained if we restrict the detection to S2 segments are presented in Table 2*.

### Impact of the amount of available training data on the performance

**Figure 7:**
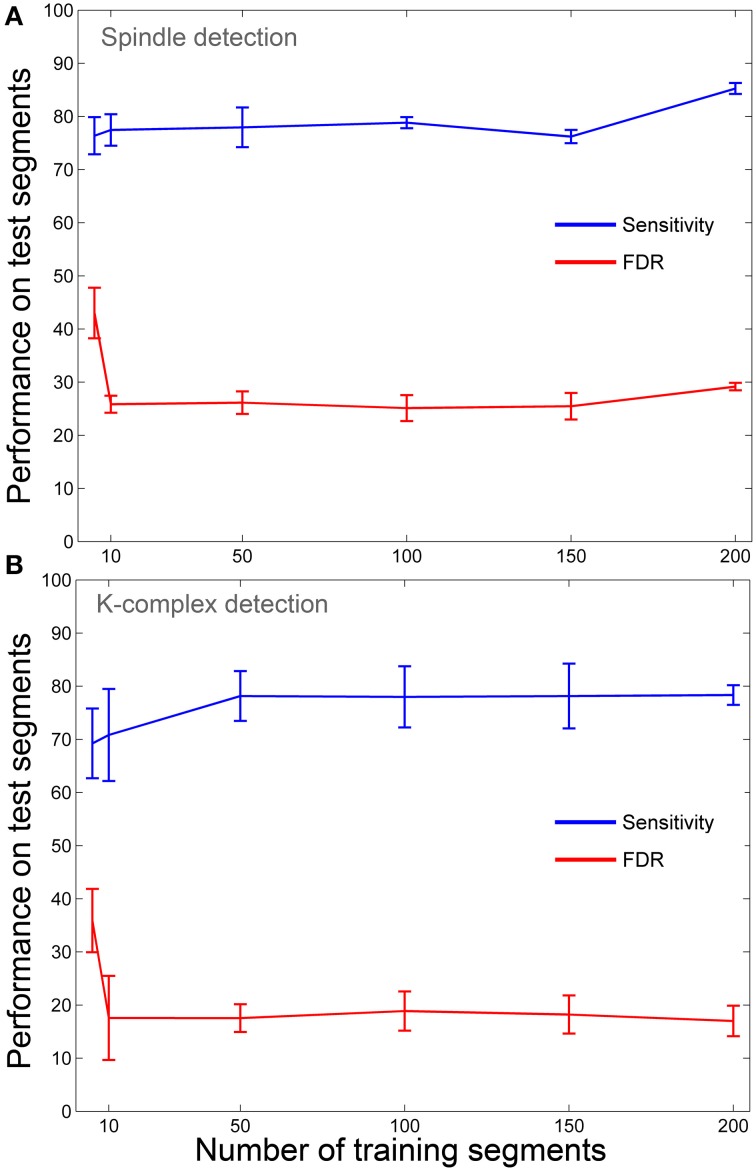
**Detection performance as a function of training set size**. **(A)** Effect of training set size on sensitivity and FDR of spindle detection. **(B)** Effect of training set size on sensitivity and FDR of K-complex detection.

The method proposed here is by definition a semi-automatic procedure since it has a built-in training step that uses visual marking of a subset of data to determine an optimal threshold that is to be used on the rest of the data. An important question is therefore: what is the minimal amount of visual scoring required by our method in order to achieve acceptable detection results? Obviously the method would be of little use, if half (or more) of the K-complexes and spindles in the data need to be marked by an expert to ensure that it works. To address this question we launched the entire pipeline (training and testing) repeatedly, each time using an increasing number of training segments (starting from five segments up to 200 segments, the procedure was repeated five times at each size with random selection of segments). The aim was to see how quickly the sensitivity and FDR metrics stabilize. Here, we restricted the analysis to all segments where the annotations of both experts were in complete agreement (consensus). This was done to ensure robustness of the annotation and because of the lengthy computational cost associated with recalculating the whole analysis for annotations from each expert. The aim here was not to assess the effect of inter-expert variability, but rather to assess the dependency of our technique on the number of training samples. The results in Figure 7 show that, luckily, the performance metrics reach a plateau already with a small number of training segments (below 20 segments for spindles and below 50 segments for K-complexes). This result indicates that the proposed method can be used with minimal visual marking.

### Performance of the method in individual subjects

The results presented so far were obtained by combining EEG sleep segments extracted from multiple subjects (*n* = 14). But how robust is the proposed method for the detection of K-complexes and spindles in each individual subject? And, in particular, how good are the performances in single subjects when only a handful of events have been visually marked and thus available for training? To address this question we launched the entire detection pipeline in each subject individually using only 15 segments for training (On average these 15 30-s segments contained 18 ± 4.3 spindles and 11.9 ± 2.3 K-complex events). In addition, as in the previous analysis (Section Impact of the Amount of Available Training Data on the Performance) we restricted the analysis to all segments where the annotations of both experts were in agreement (consensus). The results listed in Table 4 indicate a reasonably good performance in each individual. The means of the individual performances (achieved from only 18 spindles and 11.9 K-complexes on average) are in fact comparable to those achieved (see Table 2) when combining the data from all subjects and using half the data for training (210 segments, consisting of 141 K-complex and 217 spindle events). As a matter of fact, the mean sensitivity for spindle detection (i.e., 84.39%) which was obtained with very low number of training samples is slightly higher than the value achieved with half of the whole data set when combining data across individuals. The results in Table 4 confirm that individually determined thresholds provide good results and, because they were achieved with only 15 training segments, it also suggests that the proposed method does not require a lot of visual marking. Note however, that for practical reasons and for the sake of generalizability we recommend the use of a global detection threshold, just as we did in all previous sections.

**Table 4:** **Performance of the TQWT-MCA spindle and K-complex detection method in each subject with minimal training**.

	**Spindle**			**K-complex**		
	**Sens %**	**FDR%**	**Th (μV^2^)**	**Sens (%)**	**FDR (%)**	**Th (μV)**
S1	61.54	20.00	300	78.57	21.42	−68
S2	85.00	50.00	210	88.89	27.27	−72
S3	100.00	27.27	240	81.81	10.00	−80
S4	62.50	16.67	300	75.00	10.00	−82
S5	73.91	19.05	270	57.14	11.11	−94
S6	81.43	23.25	280	83.33	23.08	−74
S7	96.30	35.00	240	60.00	14.28	−78
S8	90.62	9.37	270	80.76	20.00	−94
S9	90.00	47.06	230	88.89	11.11	−66
S10	95.65	21.43	260	93.33	12.50	−62
S11	100.00	50.00	210	100.00	26.67	−70
S12	100.00	23.68	270	33.33	88.88	−78
S13	76.19	11.11	300	83.33	45.22	−98
S14	68.42	31.58	340	80.00	42.86	−54
Mean	84.39	27.53	265.7	77.45	26.04	−76.4

*Sensitivity Sen (%), FDR (%), and the optimal threshold (Th) are reported for each individual but also as mean values across the whole population (bottom row). Only 15 annotated 30-s EEG segments were used for training in each subject (corresponding on average to 18 ± 4.3 spindles and 11.9 ± 2.3 K-complex training events)*.

### Comparison with a standard detection method

To gain insights into how our method compares to existing methods, we implemented a standard spindle detection method (Gais et al., 2002; Mölle et al., 2002) which has already been implemented or used as a standard method for comparison, in numerous publications (e.g., Gais et al., 2002; Mölle et al., 2002; Bergmann et al., 2012; Feld et al., 2013; Parekh et al., 2014, 2015; Warby et al., 2014). In brief, the procedure consists of the following steps: (1) filtering the EEG with a 12–15 Hz bandpass filter, (2) calculating the root mean square (RMS) of each 100 ms interval of the filtered signal, (3) counting the number of times the RMS power crossed a constant detection threshold *T* value for 0.5–3 s. In the original study, Mölle et al. (2002) set the threshold *T* to 10 μV. To choose the best value for this parameter with regards to our data, we computed the performances we achieved using all *T* values between 5 and 12 μV (in 1 Hz steps) on the training test. The threshold that provided the best compromise between sensitivity and FDR was the one used when applying our method to the test data. Table 5 compares the results obtained with this standard method (Mölle et al., 2002) to those obtained with our method, but also to a hybrid approach where we use our TQWT + MCA analysis as a pre-processing before running the standard RMS-procedure proposed in Mölle et al. (2002). The results in Table 5 suggest that our method outperforms the RMS-based method on the same data set. In addition, we found that the performance of the RMS-based method (Mölle et al., 2002) can be substantially improved if we first apply our TQWT-MCA processing to the data. Note that the thresholds *T* that yielded the best results with our data were 6 and 8 μV for the detection with and without TQWT-MCA, respectively.

**Table 5:** **Comparison between the results (Sensitivity and FDR) achieved with our method to those obtained by applying a standard spindle detection technique (Mölle et al., 2002), and to those achieved by a hybrid approach where we use the proposed TQWT + MCA analysis as a pre-processing step before running the standard RMS-based detection procedure**.

	**Filtering + RMS (Mölle et al., 2002)**				**Current study**	
	**Standard**		**With TQWT**		**Expert 1**	**Expert2**
	**Expert 1**	**Expert 2**	**Expert 1**	**Expert 2**		
Sensitivity	70.30	70.56	74.06	75.88	83.18	83.10
FDR	49.45	46.21	42.22	37.24	39	19.66

*The performances of these three approaches are reported against Expert 1 and Expert 2 independently. The best results were achieved with the method proposed in this study*.

### Performance evaluation on a publicly available database

To investigate the performance of our method on sleep EEG data other than our own recordings, we detected spindles and K-complexes by applying our method to the DREAMS data set, a publicly available database of annotated sleep EEG. EEG recordings from two specific databases were used: The Sleep Spindle database and the K-complexes database, which have both been made available by University of MONS - TCTS Laboratory and Université Libre de Bruxelles—CHU de Charleroi Sleep Laboratory. The spindles data can be accessed online at: http://www.tcts.fpms.ac.be/~devuyst/Databases/DatabaseSpindles/ while the K-complex data can be found at: http://www.tcts.fpms.ac.be/~devuyst/Databases/DatabaseKcomplexes/. The spindles and K-complexes databases consist respectively of 8 and 10 excerpts of 30 min of annotated central EEG channel extracted from whole-night PSG recordings. Here, we used recordings from the subjects that were recorded with identical sampling rate (200 Hz) and for which the visual annotation was complete. This meant that for spindle detection we used 6 participants out of 8 and for the K-complex detection we used the data from all 10 participants. We used the annotation by Expert 1 as benchmark since the annotations of Expert 2 are not available for all subjects. The straight-forward application of our method to these data, without any specific parameter adaptations, yielded a sensitivity of 71.77% and FDR of 30.54% for spindle detection, and a sensitivity of 83.31% and FDR of 36.31% for K-complex detection.

## Discussion

The current study proposes a new method for joint detection of sleep spindles and K-complex events, two hallmarks of NREM sleep stage 2, by conveniently splitting the EEG signal into oscillatory (spindles) and transient (K-complex) components. The decomposition is achieved by applying MCA on a sparse representation of EEG segments obtained by the recently introduced discrete TQWT (Selesnick, 2011a,b,c) with parameters specifically *tuned* to spindle and K-complex characteristics. The actual detection step relies on thresholding (a) the transient component in the search for K-complexes and (b) the time-frequency representation of the oscillatory component in search for sleep spindles. Optimal thresholds are extracted from ROC-like curves (sensitivity vs. FDR) in a training set, and the performance of the method is assessed on the test set.

Overall the method presented here provides a reasonable compromise between sensitivity and FDR with performances that were robust on several levels: First, the performances did not change much when the benchmarking ground-truth was switched from one scorer to another [Section Spindle and K-complex Detection Performance (Test Set)]. Second, the performance hardly changed whether only stage2 sleep EEG segments were used or if data from all sleep stages were examined (Section Stability of the Proposed Method with Regards to Sleep Stages). Third, and most importantly, our results show that the method does not require a large training set to derive optimal cut-off thresholds. By varying the number of segments used for training, we found that the performance in terms of sensitivity and FDR reaches a plateau within less than 20 training segments (Section Impact of the Amount of Available Training Data on the Performance, Figure 7). Finally, the latter observation was further confirmed by running the detection pipeline on individual subjects where the training (search for optimal threshold) was restricted to 15 segments (i.e., using on average 18 spindles and 12 K-complexes). This analysis revealed good sensitivity and relatively low FDR in each subject and also in terms of means over all individuals (Section Performance of the Method in Individual Subjects, Table 4).

The TQWT-MCA approach has been recently used to dissociate transient events with or without high frequency oscillations (HFOs) in intracranial EEG (Chaibi et al., 2014). The current study, is to our knowledge, the first to demonstrate the utility of the TQWT-MCA framework for the detection of sleep spindles and K-complexes.

Furthermore, the results we obtained by excluding the TQWT-MCA decomposition from the proposed framework, confirmed and quantified its contribution to the high performances obtained (Section Performance Comparison with and without TQWT and MCA). Compared to the results obtained without the TQWT-MCA step, our method achieved an additional 13 point increase in percent sensitivity for spindles and a five point increase for K-complexes (Figure 6). Since the proposed decomposition is based on sparse representation of spindles and K-complexes, it reduces the effect of noise and artifacts in EEG signals, which may explain, at least in part, the improved performance of the subsequent CWT and FIR filtering.

In addition, we have shown that a simple visual marking adjustment step can lead to significant improvements, in particular by reducing FDR. In the scorer adjustment procedure the expert is presented with the false positive detections and is given the possibility to accept or reject detections that he had initially not indicated but that the algorithm identified as being positives. This SA procedure is not part of the recommended algorithm, rather a way to identify and quantify cases where the objective machine might actually outperform the subjective human scorer.

Parekh et al. (2014) propose a strategy to improve spindle detection by pre-processing the raw EEG signal using non-linear dual Basis Pursuit Denoising (BPD) which is also a way to separate the non-oscillatory transient components of the signal from the sustained rhythmic oscillations. The subsequent filtering of the oscillatory component enhances the spindles with regards to baseline, and thereby improves their detectability with standard spindle detectors. Using this technique with a readily available EEG spindle database provided a mean increase of 13.3% in the by-sample F1 score and 13.9% in the by-sample Matthews Correlation Coefficient score. A recent study by the same group also provides compelling evidence for the added value of using sparse optimization to detect spindles and K-complexes (Parekh et al., 2015). A direct comparison between these approaches and the methodology proposed here is not straightforward given the use of by-sample metrics in the Parekh et al. (2014, 2015) studies. Most importantly, the current method and those proposed by Parekh et al. (2014, 2015) provide converging evidence of improved spindle detection via time-frequency sparsity, and they collectively suggest that this framework is a promising path for enhanced performance of event detection in sleep EEG.

Overall, the results reported here (either by combining data across participants or by performing the detection algorithm separately for each individual) are comparable with the results of existing methods. However, we performed further analyses in order to gain additional insights into (a) how the performance of the pipeline proposed here compares to existing methodology (Section Comparison with a Standard Detection Method) and (b) how well it performs on other available data sets (Section Performance Evaluation on a Publicly Available Database). The results suggest that our method provides better detection than the RMS-based method and that the performance of the latter can be improved if we first apply the TQWT-MCA processing to the data before computing the RMS (Table 5). Furthermore, application of our method to the Devuyst et al. (2010, 2011) online database, yielded a sensitivity of 71.77% and FDR of 30.54% for spindle detection, and a sensitivity of 83.31% and FDR of 36.31% for K-complex detection. The original papers associated with these databases do not directly report sensitivity and FDR, but these metrics can be inferred from the confusion matrices they provided for each expert. Using Expert 1 as ground truth (as we did here), they detected spindles with sensitivity of 68.40% and FDR of 62.04% (computed from confusion matrix in Devuyst et al., 2011). As for K-complexes, they were detected with sensitivity of 61% and FDR of 26.70% (computed from confusion matrix in Devuyst et al., 2010). Note, however, that the comparison between their findings and ours is limited by the fact that the recordings provided online does not allow us to explore the exact data sets used in Devuyst et al. (2010, 2011).

More generally, the comparison between existing methods for spindle and/or K-complex identification is not an easy endeavor. First of all, the different methods proposed are generally evaluated on different EEG data sets and with different scorers, often with substantial inter-rater variability (Wendt et al., 2015). Moreover, performance metrics also tend to differ across studies. Recent efforts seek to overcome such limitations by providing free access to high quality annotated sleep EEG data sets (O'Reilly et al., 2014). Such benchmark data carry the potential to significantly advance the field of automatic spindle and K-complex detection, as well as sleep staging. This was performed in a recent report by O'Reilly and Nielsen (2015) where the authors compared four automatic spindle detection algorithms: Teager detector (Ahmed et al., 2009), Sigma index (Huupponen et al., 2007), RSP (Devuyst et al., 2011), RMS (Mölle et al., 2002). To this end, four data bases were used, two of which are open access: the DREAMS database (Devuyst et al., 2010, 2011) and the Montreal Archive of Sleep Studies (MASS) (O'Reilly et al., 2014). The results obtained and conclusions drawn from this important comparison highlight limitations and shortcomings of classical detection performance evaluations frameworks. In particular, the reported findings question the reliability of using expert scoring as gold standard. In addition, they highlight the necessity of using an exhaustive set of performance metrics: The authors recommend the use of sensitivity, precision and a more comprehensive statistic such as Matthew's correlation coefficient, F1-score, or Cohen's κ for adequate sleep spindle assessment. Comparison of our results with those presented in this comparative study is not straightforward because we use window-based performance metrics whereas the study by O'Reilly and Nielsen (2015) use a signal-sample metric, equivalent to the “by-sample” metric (Warby et al., 2014). This discrepancy is in itself problematic. Future studies should seek to evaluate detection performance using a unified set of evaluation metrics computed on large open-access benchmarking data bases. Such an assessment of the method proposed here would certainly help evaluate its strengths and limitations.

The current study is one of a few reports that have proposed a common methodological framework for the joint detection of K-complex and spindles (Jobert et al., 1992; Koley and Dey, 2012; Jaleel et al., 2013; Camilleri et al., 2014; Parekh et al., 2015). While Jobert et al. (1992) used matched filtering to detect sleep spindles and K-complex waveforms, Camilleri et al. (2014) used switching multiple models. The authors of the latter study evaluated their method by computing sensitivity and specificity based on two expert manual scores and reported a sensitivity of 83.49 and 52.02% and a specificity of 78.89 and 90.55% for respectively spindles and K-complex detection. In addition, Koley and Dey (2012) used CWTs to detect a set of sleep EEG characteristic waveform, including spindles and K-complex. They reported a good accuracy of 92.6 and 93.9% but didn't mention any performance metrics that take false positive or false negative detection into account. Jaleel et al. (2013) proposed a pilot detection method based on a mimicking algorithm which imitates human visual scoring. However, no systematic evaluation of performance metrics was provided. The method proposed by Parekh et al. (2015) provides an elegant approach based on the decomposition of the EEG signals into three signal components (low-frequency, transient and non-oscillatory) and their results highlight the utility of sparse optimization in the improved detection of spindles and K-complexes.

Because of the naturally low number of K-complexes or spindles across some of the stages (S1 and REM in particular) it was impossible for us here to conduct our detection pipeline on each sleep stage individually. Instead, we evaluated the performance of our method by using either only S2 segments, or by pooling segments from all stages (S2 and non-S2 segments). Future studies with larger annotated sleep EEG databases will be needed to assess and compare the robustness of our method in each single sleep stage.

One way to increase the performance of our method could be to fine-tune parameters of the TQWT and of the MCA procedures on a subject by subject basis, so as to account for inter-individual differences in spindle and K-complex properties. To what extent the performance can be improved by modifying the tuning Q-factor (globally or for each individual) is not clear and could be the focus of further investigation. Future explorations may also benefit from exploring the use of alternative wavelets, such as the Morse wavelet (Lilly and Olhede, 2012) which has successfully been used in recent studies (Zerouali et al., 2013, 2014; O'Reilly et al., 2015).

Moreover, it is possible that the false positive detections in our pipeline include vertex waves mistakenly identified as K-complexes since the two events bare strong resemblances. Careful selection of the FIR filter parameters may help reduce this risk since vertex waves are shorter-lived events (<0.5 s).

A further path for performance improvement is to seek to identify spindles and K-complexes in multi-electrode data. The co-occurrence (and even delays) of the presence of these micro-structure across parietal, temporal and frontal brain areas would be very informative, and could even be used to increase detection performance. In addition, exploring the results obtained with the proposed method across all scalp-EEG channels could be helpful in assessing the distribution and propagation of K-complexes and spindles (O'Reilly and Nielsen, 2014a,b) and unraveling their underlying network dynamics (Zerouali et al., 2014). Note also that the Q-factor of the TQWT can easily be tuned to incorporate differences in frequencies between, for instance, faster central spindles and slightly slower frontal spindles (e.g., Andrillon et al., 2011).

Another venue for future research would also be to attempt to incorporate into our framework recent findings of cross-frequency relationships among various electrophysiological signatures of sleep. In particular, high-frequency activity in the gamma-range, which has been shown to be involved in a variety of cognitive processes (e.g., Jerbi et al., 2009a,b; Jung et al., 2010; Dalal et al., 2011; Lachaux et al., 2012; Perrone-Bertolotti et al., 2012; Vidal et al., 2014), has also been shown to co-fluctuate with slower brain rhythms (Jensen and Colgin, 2007; Canolty and Knight, 2010; Soto and Jerbi, 2012). During sleep, gamma oscillations have been linked to spindles (e.g., Ayoub et al., 2012) and to slow wave sleep in intracranial EEG recordings (Dalal et al., 2010; Le Van Quyen et al., 2010; Valderrama et al., 2012) and in non-invasive EEG recordings (Piantoni et al., 2013). Whether including these cross-frequency relationships will enhance current detection tools remains to be seen.

## Conclusion

The current study demonstrates the feasibility of identifying spindles and K-complex events in sleep EEG using a single methodological framework by literally *tuning into* the oscillatory characteristics of the target events via the TQWT. Because of the now well acknowledged challenges that face performance evaluation of automatic and semi-automatic procedures (O'Reilly et al., 2014), the next step would be to validate our method on a larger open-access benchmarking sleep database. This would allow us to perform fair and informative comparisons with other existing methods, and possibly to fine-tune the parameter selection for our method. From a broader perspective, the flexibility with which the TQWT and MCA decomposition (Selesnick and Bayram, 2009; Selesnick, 2011a,b,c) can be *tuned* to specific oscillatory or transient phenomena in the signal suggests that it could be a promising tool for the detection of other structures in sleep EEG signals beyond those included in this study, such as vertex wave, slow waves and apnea.

### Conflict of interest statement

The authors declare that the research was conducted in the absence of any commercial or financial relationships that could be construed as a potential conflict of interest.
